# Texas as a Health Resort

**Published:** 1889-10

**Authors:** 


### TEXAS AS A HEALTH RESORT.

It is a shame, and a reproach on the greatness of Texas in all else, that despite the oft declared necessity of preservation of her vital statistics urged upon our lawmakers by the medical profession, she has no State Board of Health,—no adequate provision for getting and preserving the vast amount of material in the way of statistics, which, if properly utilized, could induce a rapid settlement and development of her millions of acres of unoccupied lands. The only provision, or attempt at it, made by the legislature to do this thing, is a feature in the office of Commissioner of Agriculture, by which he is enabled to get a partial record. In as much as we are often interrogated by letter on the subject of the healthfulness of Texas, we append below a summary from the last report of Commissioner Foster, which, so far as it goes, is a most satisfactory showing. We venture there is no country on the face of the globe with so low a rate of mortality as is here shown:

DEATHS IN TEXAS IN 1887.

| | W. M. | W. F. | C. M. | C. F. | Total |
|---|---|---|---|---|---|
| Consumption . . . . . | 454 | 527 | 114 | 206 | 1301 |
| Diarrhœa, Inf. . . . . | 374 | 249 | 94 | 56 | 773 |
| Dysentery . . . . . . | 208 | 184 | 48 | 59 | 499 |
| Pneumonia . . . . . . | 626 | 467 | 211 | 120 | 1424 |
| Typhoid Fever . . . . | 623 | 594 | 96 | 102 | 1,415 |
| Congestive Chill . . . | 407 | 295 | 63 | 62 | 827 |
| Hæmaturia . . . . . . | 79 | 53 | 10 | 13 | 155 |
| Bright's Disease . . . | 85 | 46 | 11 | 7 | 149 |
| Chill and Fever . . . | 101 | 101 | 58 | 37 | 297 |
| Scarlet fever . . . . . | 80 | 81 | 7 | 7 | 175 |
| Measles . . . . . . . . | 234 | 238 | 51 | 46 | 569 |
| Malaria . . . . . . . . | 205 | 180 | 60 | 62 | 507 |
| Sunstroke . . . . . . . | 13 | 21 | 2 | 2 | 38 |
| All other diseases . . | 5,135 | 4,884 | 1,236 | 1,431 | 12,687 |
| | 8,625 | 7,920 | 2,061 | 2,210 | 20,816 |

Estimated population, 2,500,000 in 1887.

Ratio per 1000 inhabitants, of deaths from all causes except accident, murder and suicide, 8.33.

In the 12,687 cases reported as "unclassified," doubtless are included still births, accidents, etc., which is illegitimate; hence it is fair to assume that the ratio of deaths to population for twelve months does not exceed about 7.

Under the head of consumption, are reported 1301 deaths, as follows; White males, 454; white females, 527; colored males, 114; colored females, 206. Doubtless many of these cases were chronic bronchitis, or diseases of the liver, or other diseases simulating consumption, reported as consumption frequently, no doubt by the relatives, and in the absence of a physician, for it must be remembered that in the sparsely settled districts there are few physicians; and again, there is abundant reason to believe that most of the deaths from real consumption occurred in persons who had come into Texas with the disease, attracted here by the mild climate and the well-known curative properties of the Texas atmosphere for many pulmonary diseases, hence a big allowance must be made on that score; while it will be seen that the number of deaths from diseases usually attended with the greatest mortality elsewhere is remarkably small, and not a death is reported from diphtheria, yellow fever, small-pox, cholera, etc., etc.

